# Ultrasonography of the Vagus Nerve for ALS Patients: Correlations with Clinical Data and Dysfunction of the Autonomic Nervous System

**DOI:** 10.3390/medicina61050902

**Published:** 2025-05-16

**Authors:** Ovidijus Laucius, Justinas Drūteika, Tadas Vanagas, Renata Balnytė, Andrius Radžiūnas, Antanas Vaitkus

**Affiliations:** 1 Department of Neurology, Medical Academy, Lithuanian University of Health Sciences, 44307 Kaunas, Lithuania; justinas.druteika@lsmu.lt (J.D.); renata.balnyte@lsmu.lt (R.B.); antanas.vaitkus@lsmu.lt (A.V.); 2 Department of Neurosurgery, Medical Academy, Lithuanian University of Health Sciences, 44307 Kaunas, Lithuania; andrius.radziunas@lsmu.lt

**Keywords:** vagus nerve, ultrasound, heart rate variability, ALS, autonomic nervous system

## Abstract

*Background and Objectives:* Amyotrophic lateral sclerosis (ALS) is a progressive neurodegenerative disorder characterized by the degeneration of both upper and lower motor neurons, leading to the rapid decline of motor function. In recent years, dysfunction of the autonomic nervous system (ANS) has also been increasingly recognized as a contributing factor in various neurodegenerative diseases, including ALS. This study is the second publication from our ALS research cohort at Kaunas Clinics. Our previous work examined ultrasonographic changes in the phrenic nerve as a supplementary diagnostic approach for ALS. *Materials and Methods:* In the present study, we investigated ultrasonographic alterations of the vagus nerve within the same ALS cohort, aiming to explore correlations with ANS involvement. We performed high-resolution ultrasonography of the vagus nerve (VN), collected clinical data, conducted heart rate monitoring, and evaluated respiratory function. *Results:* We prospectively included 32 ALS patients meeting “Gold Coast” criteria and 64 age- and sex-matched control patients. The average onset of ALS was 57.97 ± 9.22 years, and the duration of the disease was15.41 ± 9.04 months. For ALS patients, we found significantly reduced vagus nerve cross-sectional area (CSA) at the level of the carotid artery bifurcation bilaterally compared to controls (right VN 1.86 ± 0.21 vs. 2.07 ± 0.18 mm^2^, *p* < 0.001; left VN 1.69 ± 0.21 vs. 1.87 ± 0.21 mm^2^, *p* < 0.001). Reduced values of the left VN positively correlated with the reduced values of FEV1% and sO_2_. *Conclusions:* Our findings revealed a significant bilateral reduction in vagus nerve size in ALS patients compared to controls, suggesting that vagal atrophy may serve as a potential marker of autonomic dysfunction in ALS.

## 1. Introduction

Amyotrophic lateral sclerosis (ALS) is a progressive neurodegenerative disease marked by muscle weakness and paralysis, ultimately resulting in fatality due to the degeneration of both upper and lower motor neurons [1,2,3]. The exact cause of ALS remains largely unknown in most cases. Approximately 90–95% of ALS cases are sporadic, while genetic factors account for 5–10% [4,5]. A small percentage of ALS cases are familial and are associated with mutations in genes such as SOD1, C9orf72, and TARDBP [6].

While electroneuromyography (ENMG) is the gold standard for diagnosing ALS, its sensitivity is not absolute and may vary depending on the operator’s expertise. This limitation is particularly pronounced in ALS cases with predominant upper motor neuron (UMN) involvement, especially those with initial bulbar onset. Nonetheless, ENMG remains valuable in detecting subclinical lower motor neuron (LMN) involvement, providing critical evidence to support a clinical diagnosis of ALS [7]. The time to ALS diagnosis is largely influenced by the initial symptom presentation, which can vary depending on the affected anatomical region, such as the bulbar, limb, axial, or respiratory systems. This variability often necessitates differential diagnosis, specialized evaluations, and, in some cases, consultation with non-neurological specialists, which can delay the diagnostic process and adversely impact patient outcomes [8]. Efforts to develop faster, more cost-effective, and accessible diagnostic methods are ongoing.

Several studies have demonstrated the degeneration of peripheral nerves, including the vagus [9,10,11], phrenic [12], and peripheral limb nerves [13], during the progression of ALS. Recent studies indicate that skin innervation alterations, including a progressive reduction in Meissner corpuscles and intraepidermal nerve fibre changes, correlate with ALS progression and may serve as a valuable prognostic biomarker [14]. High-resolution ultrasound studies have detected significant atrophy of the VN in patients exhibiting bulbar symptoms, suggesting degeneration of motor fibres within the nerve [10]. This atrophy is believed to result from the degeneration of motor neurons in the brainstem nuclei, such as the nucleus ambiguus, which are responsible for innervating muscles involved in swallowing and speech [10]. Consequently, atrophy of the VN may contribute to bulbar dysfunctions commonly seen in ALS, including dysphagia and dysarthria [15].

Emerging research highlights the potential influence of body composition on peripheral nerve structure and function. Greater muscle mass and its associated metabolic activity may influence local blood flow and the neural microenvironment, potentially contributing to alterations in nerve architecture [16]. These findings indicate that metabolic and mechanical factors, beyond the primary neurodegenerative processes, might affect peripheral nerve integrity in ALS. Consequently, assessing peripheral nerve structure and composition could provide additional insights into ALS pathophysiology and help identify potential biomarkers for earlier diagnosis. In conjunction with autonomic dysfunction [17]—manifested by cardiac abnormalities, respiratory and gastrointestinal disturbances, urinary tract issues, and erectile dysfunction—these findings suggest that peripheral and autonomic nervous system involvement may precede the more prominent muscle atrophy typically observed by patients, clinicians, and through ENMG recordings.

This study is the second publication from our ALS research cohort at Kaunas Clinics. Our previous work examined ultrasonographic changes in the phrenic nerve as a supplementary diagnostic approach for ALS [12]. In the present study, we investigated ultrasonographic alterations of the VN within the same ALS cohort and added additional results from the assessment of respiratory function and heart rate monitoring, which were not done previously. Our aim was to explore changes of the VN in ALS and find possible correlations with the involvement of the ANS.

## 2. Materials and Methods

The study was conducted at the Department of Neurology, Lithuanian University of Health Sciences, with ALS patients recruited between 2022 and 2023. Participants were categorized into two groups: the ALS group (N = 32), consisting of individuals meeting the “Gold Coast” diagnostic criteria for ALS, and a control group (N = 64).

Comprehensive clinical and demographic data were collected through interviews and standardized questionnaires, including information on age, gender, height, body mass index (BMI), hip and waist circumference, disease duration, comorbidities, and medication use. In ALS patients, ultrasonography of the VN (USVN) was performed to assess its cross-sectional area (CSA), homogeneity, and echogenicity. These morphological parameters were analysed in relation to clinical features, respiratory function tests (RFTs), arterial blood gas (ABG) analyses, heart rate variability (HRV) analysis, and standardized assessments, including the revised ALS Functional Rating Scale (ALSFRS-R) and the Composite Autonomic Symptom Scale 31 (COMPASS-31). Age- and gender-adjusted normative values for ABG and RFTs were referenced from published literature. ENMG was conducted in all ALS patients to confirm the diagnosis based on the “Gold Coast” criteria [18].

Control group patients were selected randomly and underwent USVN. Inclusion criteria required all control participants to be over 18 years of age and free from neurodegenerative diseases, polyneuropathies, neuromuscular junction disorders, endocrine disorders, oncological diseases, or any conditions affecting respiratory function. Additionally, none of the control participants were on medications known to influence respiration or heart rate.

### 2.1. Ultrasonography of the Vagus Nerve (USVN)

The USVN was performed by two investigators using a high-resolution Philips EPIQ 7 ultrasound system equipped with a linear 4–18 MHz transducer (CE 0086). The transducer was positioned transversely above the clavicle in the cervical region, aligning with the level of the levator scapulae muscle where the VN courses along the anterior scalene muscle. In B-mode ultrasonography, the VN was visualized adjacent to the carotid artery bifurcation, posterior to the confluence of the internal and common carotid arteries. The nerve was identified within its connective tissue sheath, exhibiting a characteristic sonographic appearance with a centrally hypoechoic region surrounded by a hyperechoic periphery.

VN diameter was measured in millimetres using cross-sectional imaging at two predefined locations: near the carotid bulb and at the junction where the common carotid artery bifurcates. Both quantitative and qualitative characteristics of the nerve were evaluated, including its structural homogeneity (classified as homogeneous or heterogeneous) and echogenicity (categorized as hypoechoic, isoechoic, or hyperechoic).

**Figure 1:**
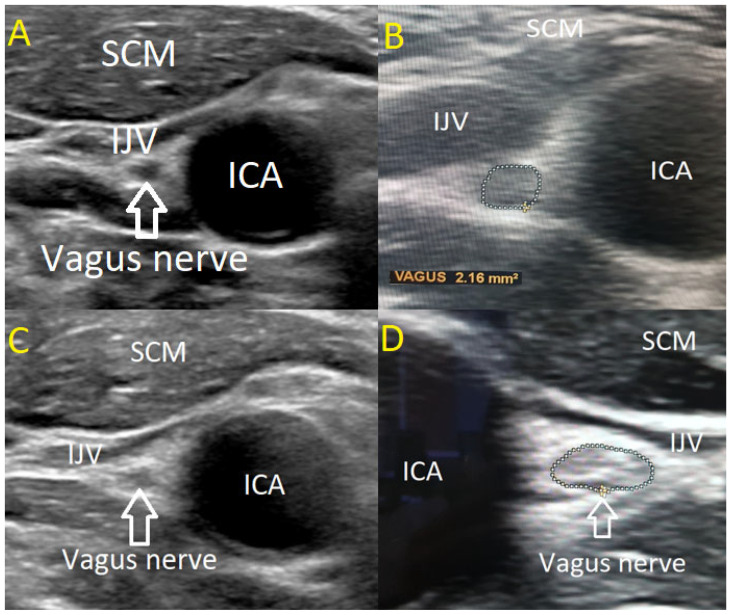
(**A**) Normal results of the USVN in a healthy volunteer (hypoechoic, homogenic). (**B**) Methodology for measuring CSA of the VN by ultrasound (isoechoic, homogenic). (**C**) USVN of ALS patient (hyperechoic, homogenic). (**D**). USVN of ALS patient (hyperechoic, heterogeneous). SCM—sternocleidomastoid muscle. IJV—internal jugular vein. ICA—internal carotid artery.

The CSA of the VN was determined in a transverse plane by tracing the hypoechoic nerve region within the hyperechoic border, following the methodology established by Walter et al. in 2018 [19]. Measurements were obtained independently by two examiners, each assessing one side three times with a precision margin of 0.01 mm^2^. The mean of the three measurements was calculated for each side, followed by a grand average across both sides. To ensure measurement reliability, both examiners were blinded to each other’s results. The measuring methodology and VN anatomical structure are illustrated in Figure 1.

### 2.2. Respiratory Function (RF)

To assess RF in ALS patients, participants were referred for a pulmonology consultation. During this evaluation, all individuals underwent a chest X-ray, ABG analysis, and spirometry. The chest X-ray was performed to assess lung structure, while ABG analysis measured key respiratory parameters, including arterial pH, partial pressures of oxygen (pO_2_) and carbon dioxide (pCO_2_), base excess (BE), bicarbonate concentration (HCO_3^−^_), and oxygen saturation (sO_2_).

Spirometry was conducted to determine forced vital capacity (FVC) and forced expiratory volume in one second (FEV1), adhering to the 2022 European Respiratory Society (ERS) and American Thoracic Society technical standards for lung function assessment. The pulmonologist analysed the results and provided a clinical interpretation of respiratory function.

### 2.3. Heart Rate Variability (HRV)

Autonomic dysfunction in ALS can present as sympathetic hyperactivity and sympathovagal imbalance, manifesting at both early and advanced disease stages [20]. In severe cases, this dysfunction may lead to critical autonomic disturbances, such as autonomic storms, characterized by profound fluctuations in blood pressure and heart rate, potentially resulting in sudden death.

HRV is a key physiological parameter used to assess fluctuations in the time intervals between consecutive heartbeats, providing valuable insights into neurocardiac function. It serves as an indicator of the heart’s ability to adapt to physiological and environmental changes through dynamic interactions between the heart and brain via the ANS [21]. Rather than being solely a measure of cardiac function, HRV reflects the complex interplay of multiple regulatory systems operating on different timescales, allowing the organism to adapt to environmental and psychological fluctuations. HRV serves as a crucial marker of autonomic regulation, influencing essential physiological functions such as blood pressure control, respiratory gas exchange, and vascular tone modulation. Additionally, it extends beyond cardiovascular control, impacting the gastrointestinal and possibly musculoskeletal systems, thereby providing a broad reflection of autonomic nervous system coherence [22].

In this study, HRV was calculated using 24-h heart rate monitoring data obtained from ALS patients wearing Holter monitors. Three primary HRV parameters were analysed: SDNN, RMSSD, and pNN50 [22]. SDNN, the standard deviation of normal sinus beat interbeat intervals, is considered the gold standard for medical stratification of cardiac risk. RMSSD, the root mean square of successive differences between normal heartbeats, represents short-term variations in heart rate and is the main time-domain metric used to assess vagally mediated fluctuations in HRV. Lastly, pNN50, the percentage of adjacent NN intervals differing by more than 50 milliseconds (ms), is closely correlated with parasympathetic nervous system activity.

Research on early-stage ALS, particularly cases involving bulbar onset, has identified significant autonomic alterations even at rest [23]. These findings suggest that autonomic dysfunction may develop early in ALS, particularly affecting parasympathetic cardiovascular control. This is evidenced by reduced overall and high-frequency HRV power, indicating impaired sinus arrhythmia, as well as altered HRV responses during tilt-table testing [24]. Such observations highlight autonomic dysregulation as an early hallmark of ALS, potentially preceding significant motor impairment.

### 2.4. Composite Autonomic Symptom Scale 31

The Composite Autonomic Symptom Scale 31 (COMPASS-31) represents a significant advancement in the assessment of autonomic dysfunction. This tool efficiently condenses the extensive 169-item Autonomic Symptom Profile (ASP) into a streamlined 31-question instrument, maintaining its effectiveness in evaluating autonomic symptoms across six domains [25]. Notably, the COMPASS-31 has shown promise in distinguishing Multiple System Atrophy with predominant parkinsonism (MSA-P) from Parkinson’s Disease (PD) [26]. A study conducted by Kim et al. demonstrated that COMPASS-31 is particularly effective in evaluating autonomic dysfunction in patients with parkinsonism. The study highlighted its significant correlation with objective autonomic function test results, showcasing its ability to differentiate MSA-P from PD with high sensitivity and moderate specificity. This distinction was especially pronounced in drug-naive patients, underscoring the tool’s potential in early diagnosis. The COMPASS-31′s design allows it to retain the comprehensive nature of the original ASP while reducing respondent burden, making it a more user-friendly option for both clinical and research purposes.

## 3. Statistical Analysis

Statistical analysis was conducted using both descriptive and comparative methods with Microsoft Excel and IBM SPSS 29.0 software. For the comparison of categorical data in the tables, the Chi-squared χ^2^ test was used. Correlation analyses were carried out using Pearson’s correlation test for normally distributed data and Spearman’s correlation test for non-normally distributed data. Statistical significance was determined at a threshold of *p* < 0.05.

## 4. Results

### 4.1. Demographic and Clinical Data

In this study, we included 32 ALS patients and 64 control group patients. ALS patients were classified into three subgroups: Eighteen (56.3%) with lower motor neuron (LMN) predominance, five (15.6%) with upper motor neuron (UMN) predominance, and nine (28.1%) with the bulbar form (Table 1). ALS and control group patients were age- and sex-matched (age—59.34 ± 9.93 and 60.84 ± 10.67 respectively, *p* = 0.508, male to female ratio—1:1.37 and 1:1.28 accordingly, *p* = 0.193). The average onset of ALS was 57.97 ± 9.22 years, and the duration of the disease was 15.41 ± 9.04 months.

### 4.2. USVN

In the control group, the mean CSA of the VN was 2.07 ± 0.18 mm^2^ on the right side and 1.87 ± 0.21 mm^2^ on the left side. In contrast, ALS patients exhibited a significant reduction in the CSA of the VN, with measurements of 1.86 ± 0.21 mm^2^ on the right side (*p* < 0.001) and 1.69 ± 0.21 mm^2^ on the left side (*p* < 0.001) compared to the control group (Table 2).

Bilateral changes in homogeneity and echogenicity were observed. On the right side, 62.5% of nerves displayed heterogeneity, 50.0% were isoechoic, and 28.1% were hyperechoic. On the left side, 62.5% of nerves showed heterogeneity, 34.4% were isoechoic, and 40.6% were hyperechoic (Table 3, Table 4 and Table 5).

**Figure 2:**
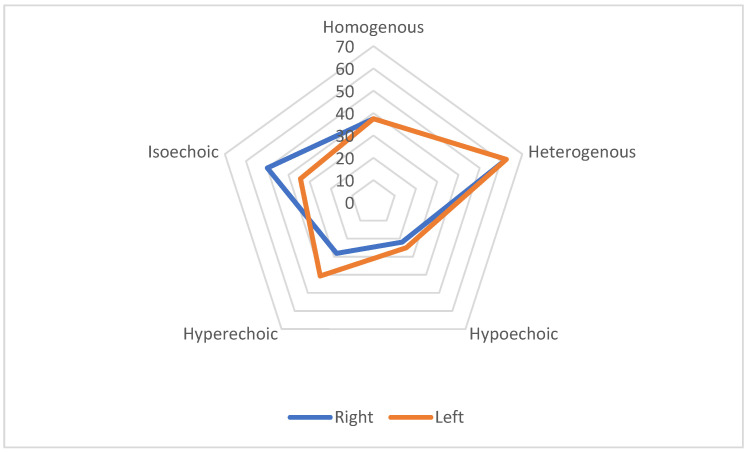
Radar chart showing homogeneity and echogenicity of the VN in ALS patients.

The radar chart in Figure 2 represents a greater number of heterogeneous than homogeneous, and hyperechoic than hypo- or isoechoic vagus nerves that were found in ALS patients.

These findings suggest potential structural alterations in the VN. The observed variations in echogenicity levels between the control group and ALS patients may offer valuable insights into the impact of the disease. Notably, most changes in homogeneity and echogenicity were predominantly observed in patients with LMN and bulbar forms of ALS, with UMN forms being less informative in this context.

### 4.3. USVN and Clinical Features of ALS Patients

In our study, USVN findings were not statistically significant when compared to the ALFRS-R scale. However, significant correlations were observed between both USVN assessments and the COMPASS-31 scale. Specifically, the correlations were significant on the right side (*p* = 0.01) and left side (*p* = 0.02).

### 4.4. VN and RF in Patients with ALS

In the evaluation of the VN and RF, USVN of the right side of the VN did not reveal significant differences in respiratory parameters, including FEV, FEV1%, FVC, FVC%, FEV1/FVC ratio, and FEV1/FVC%. However, on the left side, significant changes were noted in the FEV1% parameter. No significant changes were observed in the other measures (Table 6).

ABG analysis, including parameters such as pH, sO_2_, sCO_2_, pO_2_, and HCO_3^−^_, was performed alongside the USVN. The only significant correlation was noted comparing sO_2_ values with the reduced CSA of the left VN for ALS patients (*p* = 0.049). No other significant correlations were found (Table 7).

### 4.5. Vagus Nerve and Heart Rate Variability (HRV)

SDNN values of ALS patients were 70.91 ± 20.39 milliseconds (ms), indicating that they all probably had compromised cardiac health. RMSSD values for ALS patients were 28.94 ± 13.38 ms. The result of pNN50 among ALS patients was extremely low, at 2.61 ± 1.83%. Usually, lower values (below 10%) may suggest reduced HRV, which could be associated with stress, aging, or poor cardiovascular health. No correlations were found between HRV and the cross-sectional areas of right or left VN (Table 8).

## 5. Discussion

This study represents the first investigation of its kind in Lithuania. We performed a comparative analysis of the VN, evaluating its CSA, homogeneity, and echogenicity. These morphological parameters were assessed alongside clinical indicators, RF tests, ABG analyses, and clinical assessments using the ALSFRS-R and COMPASS-31 scales. This comprehensive approach allowed us to explore the correlation between USVN characteristics and various clinical and functional parameters in ALS patients, particularly focusing on respiratory function and heart rate variability.

This study revealed significant morphological changes in the VN of ALS patients compared to healthy controls. The observed reduction in the CSA of the VN in ALS patients highlights the nerve’s susceptibility to neurodegenerative processes. Notably, variations in echogenicity and homogeneity suggest underlying structural alterations, potentially reflecting the severity and progression of ALS. These findings are consistent with those of other authors who have also observed reductions in the VN [9,10,11,15]. However, some researchers have not identified any ultrasonographic changes of the VN [27].

The diagnostic accuracy of the CSA of the VN alone for distinguishing bulbar from non-bulbar ALS appears to be limited, as suggested by previous studies [28]. Given these limitations, a combined diagnostic approach integrating USVN with other autonomic markers, such as HRV and composite autonomic symptom scores, could enhance its clinical utility. Additionally, alternative ultrasound parameters, such as VN fascicular structure and echogenicity, may provide a more comprehensive assessment of autonomic involvement in ALS. Further research is needed to validate these multimodal approaches and determine their role in ALS diagnostics.

The study demonstrated significant ultrasonographic associations between the right and left VN and various body metrics, including height, weight, BMI, waist circumference, and hip circumference (*p* < 0.001 for most metrics). These findings suggest that larger body size, which typically indicates greater muscle and fat mass, might be correlated with changes in vagus nerve morphology. However, control group patients with a lower mass index did not show any USVN changes in the CSA.

The observed variations in echogenicity levels between the control group and ALS patients may offer valuable insights into the impact of the disease. Notably, most changes in homogeneity and echogenicity were predominantly observed in patients with LMN and bulbar forms of ALS, with UMN forms being less informative in this context.

Our results also showed significant associations between USVN findings and the COMPASS-31 scores. This indicates that changes in VN morphology correlate with autonomic dysfunction as measured by the COMPASS-31, suggesting that changes in nerve structure may manifest in measurable autonomic symptoms in ALS patients, highlighting the complex interplay between neurological and functional aspects of the disease. The COMPASS-31 correlation with VN morphology may reflect the involvement of autonomic pathways in ALS, particularly given the role of the VN in regulating autonomic functions. The observed relationship could indicate early autonomic dysfunction even in the absence of significant structural degeneration.

However, ALSFRS-R scores did not show a similar pattern of significant correlations, except for a marginal association with left-side findings (*p* = 0.125). This suggests that while VN changes are linked with autonomic symptoms, they may not directly correlate with broader functional impairments captured by the ALSFRS-R.

Interestingly, the age at disease onset demonstrated a significant correlation with the left VN findings, but not with the right-side VN. This could indicate a side-specific vulnerability of the vagus nerve to early degenerative changes in ALS, or it might reflect asymmetrical disease progression, which is not uncommon in this condition. It could also suggest that the left VN is more significantly impacted by the disease. Additionally, the duration of illness showed a significant association with the left VN, suggesting that longer disease duration might be linked to more pronounced ultrasonographic changes, particularly on the left side.

Examination of RF was almost unremarkable, except for a significant correlation between the reduction of the left VN and FEV1% parameter, highlighting a potential asymmetrical involvement of the VN in ALS as well. The observed significant variation in FEV1% on the left VN measurements might be attributed to the different impact of ALS on nerve function, which could be associated with atrophy, potentially influencing respiratory outcomes. However, ABG analysis showed no significant correlation with the VN assessments, probably suggesting that there is no direct association between the VN characteristics and oxygen saturation in the blood.

Research in ALS has highlighted the significance of HRV in understanding the disease’s autonomic dysfunction. Studies consistently show that ALS patients exhibit significant alterations in HRV, pointing to ANS involvement, particularly in parasympathetic cardiovascular regulation. For example, ALS patients tend to demonstrate reduced HRV compared to healthy individuals, indicating vagal-sympathetic imbalance [24]. This imbalance is characterized by an increased mean resting heart rate, reduced standard deviation of R-R intervals, and an elevated low-frequency to high-frequency (LF/HF) ratio, all suggestive of altered sympathetic and parasympathetic control of cardiac function [29]. Further investigations have explored HRV variations in ALS patients with different levels of respiratory function. Advanced chaotic global analysis methods revealed increased complexity in HRV among ALS patients, particularly through chaotic parameters and entropy measures [29]. This increased HRV complexity underscores the profound impact of ALS on the ANS, extending beyond its well-known motor dysfunction. In our study, HRV analysis in the ALS group revealed reduced values of SDNN, RMSSD, and pNN50, consistent with the probable involvement of the ANS during ALS progression. However, HRV changes did not correlate with alterations in the USVN, suggesting that vagal autonomic dysfunction in ALS may be more functional than structural, aligning with previous findings that vagal visceral branches remain intact in advanced ALS stages [15,24]. Although degeneration of the VN in ALS may not be severe enough to directly impair heart rate regulation, cardiac function is modulated by a broader network of sympathetic and parasympathetic fibres within the ANS. Nevertheless, HRV alterations remain evident in ALS patients, reinforcing the role of autonomic dysfunction in disease progression.

The limitations of our study primarily include a small sample size and the lack of respiratory and cardiac function data for control participants, as such assessments are not routinely performed in healthy individuals. Secondly, ultrasonography of the peripheral nerves is challenging, and the real diagnostic accuracy might be low compared to other techniques, e.g., magnetic resonance imaging. A recent study explored high-resolution ultrasound and magnetic resonance microscopy for the differentiation of upper extremity peripheral nerve fascicles and concluded that ultrasound is less accurate [30]. The fascicular anatomy of the VN is complex and varies between individuals and along its length, which is a factor in correctly measuring CSA and requires experience. Thirdly, we did not observe the expected significant changes in HRV and did not conduct HRV analysis in the control group. A comprehensive evaluation of cardiac function was not performed, making it challenging to determine whether ALS patients were sufficiently homogeneous in this regard or if some individuals were more severely affected than others. Future studies could benefit from a multivariate analysis integrating various potential ALS diagnostic methods, such as ultrasonographic assessment of the vagus and phrenic nerves, RF tests, and HRV analysis, to identify the most reliable biomarkers.

## 6. Conclusions

Our findings underscore the complexity of neural involvement in ALS, as evidenced by alterations in USVN characteristics. However, the direct correlation between these changes and functional impairments remains variable. This complexity is further highlighted by the asymmetrical effects observed between the left and right sides of the body, suggesting a non-uniform progression of ALS. The study provides important insights into the involvement of the ANS, reflected through RF, HRV, and VN changes in ALS. Nevertheless, the diagnostic value of these markers for early ALS detection remains uncertain. Larger, prospective studies are required to test the hypothesis that these markers, particularly the degeneration of the VN and reduction in HRV, could aid in the earlier diagnosis of the disease.

## Figures and Tables

**Table 1:** Distribution of ALS patients by the predominant clinical form and sex.

ALS Form\Sex	Female	Male	All ALS
Overall, n (%)	14 (43.8%)	18 (56.3%)	32 (100%)
UMN, n (%)	3 (60.0%)	2 (40.0%)	5 (15.6%)
LMN, n (%)	5 (35.7%)	13 (72.2)	18 (56.3%)
Bulbar/pseudobulbar,			
n (%)	6 (66.7%)	3 (33.3%)	9 (28.1%)
Duration of illness, months ± min/max	15.43 ± [6–30]	15.39 ± [5–44]	15.41 ± [5–44]

Values are reported using absolute values and percentage. ALS—amyotrophic lateral sclerosis; LMN—lower motor neuron; UMN—upper motor neuron.

**Table 2:** Cross-sectional area of the VN between groups.

Side of the VN	Control Group	ALS Group	*p* Value
Right VN	2.07 ± 0.18	1.86 ± 0.21	<0.001
Left VN	1.87 ± 0.21	1.69 ± 0.21	<0.001

Values are reported as mean ± SD; χ^2^ was used to test for equality of proportions.

**Table 3:** Ultrasonographic characteristics of the right VN between groups.

Ultrasonography Characteristics		Control Group	ALS Group	*p* Value
Homogeneity	Homogeneous	62 (96.9%)	12 (37.5%)	<0.001
	Heterogeneous	2 (3.1%)	20 (62.5%)	<0.001
Echogenicity	Hypoechogenic	64 (100.0%)	7 (21.9%)	<0.001
	Isoechogenic	0	16 (50.0%)	<0.001
	Hyperechogenic	0	9 (28.1%)	<0.001

Values are reported using absolute values and percentages; χ^2^ was used to test for equality of proportions.

**Table 4:** Ultrasonographic characteristics of the left VN between groups.

Ultrasonographic Characteristics		Control Group	ALS Group	*p* Value
Homogeneity	Homogeneous	64 (100.0%)	12 (37.5%)	<0.001
	Heterogeneous	0	20 (62.5%)	<0.001
Echogenicity	Hypoechogenic	64 (100.0%)	8 (25.0%)	<0.001
	Isoechogenic	0	11 (34.4%)	<0.001
	Hyperechogenic	0	13 (40.6%)	<0.001

Values are reported using absolute values and percentages; χ^2^ was used to test for equality of proportions.

**Table 5:** Correlations of the results of USVN and the clinical features of ALS patients.

Clinical Features	Mean ± SD	Right VN US	Left VN US
Height, cm	170.43 ± 9.57	*p* = 0.001	*p* = 0.036
Weight, kg	77.95 ± 15.33	*p* < 0.001	*p* < 0.001
BMI, kg/m^2^	26.78 ± 4.39	*p* < 0.001	*p* < 0.001
Waist circumference, cm	90.23 ± 12.26	*p* < 0.001	*p* = 0.001
Hip circumference, cm	101.78 ± 10.38	*p* < 0.001	*p* < 0.001
Age, years	60.34 ± 10.39	*p* = 0.737	*p* = 0.169
Age at disease onset, years	57.97 ± 9.22	*p* = 0.468	*p* = 0.011
Duration of illness, months	15.41 ± 9.04	*p* = 0.526	*p* = 0.021
Compass31	8.89 ± 12.69	*p* = 0.01	*p* = 0.02
ALFSR-R	38.41 ± 5.86	*p* = 0.125	*p* = 0.757
Sex	-	*p* = 0.777	*p* = 0.921

Values are reported as mean ± SD; correlations were assessed using Pearson’s or Spearman’s tests, as appropriate.

**Table 6:** Correlations between measurements of the vagus nerve and respiratory parameters.

Resp. Parameters	Mean ± SD	Correlations with Right VN	Correlations with Left VN
FEV1	2.46 ± 1.07	0.617	0.064
FEV1%	75.84 ± 24.02	0.640	0.041
FVC1	3.02 ± 1.29	0.762	0.372
FVC%	73.03 ± 23.38	0.827	0.082
FEV1/FVC ratio	81.07 ± 6.32	0.600	0.489
FEV1/FVC%	102.38 ± 7.71	0.970	0.652

Values are reported as mean ± SD; correlations were assessed using Pearson’s or Spearman’s tests, as appropriate. Statistical significance: *p* < 0.05; FEV—Forced Expiratory Volume; FVC—Forced Vital Capacity.

**Table 7:** Correlations between measurements of the VN and ABG parameters.

ABG Parameters	Mean ± SD	Correlations with Right VN	Correlations with Left VN
pH	7.43 ± 0.03	0.478	0.054
pCO_2_	37.41 ± 5.45	0.364	0.261
pO_2_	94.4 ± 17.29	0.736	0.625
sO_2_	96.34 ± 1.79	0.454	0.049
HCO_3^−^_	24.03 ± 2.87	0.161	0.898
BE	0.38 ± 2.34	0.059	0.278

Values are reported as mean ± SD, correlations were assessed using Pearson’s or Spearman’s tests, as appropriate. Statistical significance: *p* < 0.05; pH—acidity scale, sO_2_—oxygen saturation; sCO_2_—carbon dioxide saturation, pO_2_—partial pressure of oxygen, HCO_3^−^_—bicarbonate concentration, BE—base excess.

**Table 8:** Correlations between measurements of the VN and HRV parameters.

HRV Parameters	Mean ± SD	Correlations with Right VN	Correlations with Left VN
SDNN, ms	70.91 ± 20.39	0.975	0.681
RMSSD, ms	28.94 ± 13.38	0.213	0.358
pNN50, %	2.61 ± 1.83	0.741	0.219

Values are reported as mean ± SD; correlations were assessed using Pearson’s or Spearman’s tests, as appropriate. Statistical significance: *p* < 0.05; SDNN—standard deviation of the interbeat intervals of normal sinus beats, RMSSD—the root mean square of successive differences between normal heartbeats, pNN50 is the proportion of NN50 divided by the total number of NN (R-R) intervals.

## Data Availability

The original contributions presented in the study are included in the article; further inquiries can be directed to the corresponding authors.
